# Optimizing in vitro T cell differentiation by using induced pluripotent stem cells with GFP‐RUNX1 and mCherry‐TCF7 labelling

**DOI:** 10.1111/cpr.13661

**Published:** 2024-06-10

**Authors:** Yu Zhao, Jiani Cao, Haoyu Xu, Weiyun Cao, Chenxi Cheng, Shaojing Tan, Tongbiao Zhao

**Affiliations:** ^1^ State Key Laboratory of Stem Cell and Reproductive Biology Institute for Stem Cell and Regeneration, Institute of Zoology, Chinese Academy of Sciences Beijing China; ^2^ University of Chinese Academy of Sciences Beijing China; ^3^ Beijing Institute for Stem Cell and Regenerative Medicine Beijing China

## Abstract

In vitro T‐cell differentiation from pluripotent stem cells (PSCs) could potentially provide an unlimited source of T cells for cancer immunotherapy, which, however is still hindered by the inefficient obtaining functionally‐matured, terminally‐differentiated T cells. Here, we established a fluorescence reporter human induced pluripotent stem cell (iPSC) line termed TCF7^mCherry^RUNX1^GFP^, in which the endogenous expression of RUNX1 and TCF7 are illustrated by the GFP and mCherry fluorescence, respectively. Utilizing TCF7^mCherry^RUNX1^GFP^, we defined that the feeder cells incorporating CXCL12‐expressing OP9 cells with DL4‐expressing OP9 cells at a 1:3 ratio (OP9‐C1D3) significantly enhanced efficiency of CD8^+^ T cell differentiation from PSCs. Additionally, we engineered a chimeric antigen receptor (CAR) targeting EGFR into iPSCs. The CAR‐T cells differentiated from these iPSCs using OP9‐C1D3 feeders demonstrated effective cytotoxicity toward lung cancer cells. We anticipate this platform will help the in vitro HSPC and T cell differentiation optimization, serving the clinical demands of these cells.

## Graphical abstract

Indicates the cell differentiation process utilizing the TCF7^mCherry^RUNX1^GFP^ reporter system.

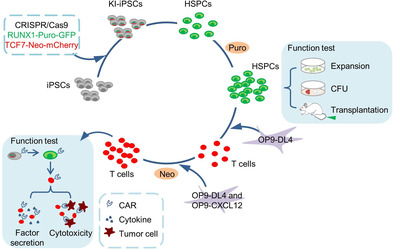

## 1 INTRODUCTION

T cell mediated immunotherapies, such as chimeric antigen receptor T (CAR‐T) and T cell receptor T (TCR‐T) therapies, have demonstrated promising results in the treatment of both haematopoietic malignancies and solid tumours.^1^, ^2^, ^3^, ^4^, ^5^, ^6^, ^7^ Typically, isolated peripheral T cells, whether autologous or allogenic, are used for clinical administration. However, these cells have limited in vitro expansion potential and tend to become exhausted after cell culture. Pluripotent stem cells (PSCs), including embryonic stem cells (ESCs) and induced pluripotent stem cells (iPSCs), offer a potential solution. These cells can proliferate indefinitely and maintain the ability to differentiate into any cell type. This makes them an appealing source for obtaining an unlimited supply of haematopoietic stem and progenitor cells (HSPCs) and T cells in vitro.^8^, ^9^, ^10^, ^11^

During the process of T cell development in the thymus, the activation of Notch signalling by Delta‐like Notch ligands, expressed in the thymic niche, plays a pivotal role in determining T‐cell fate. The Notch signalling pathway inhibits the B cell differentiation potential of lymphocyte precursor cells and directs them toward a T cell fate in vivo.^12^ Traditional methods for differentiating PSCs into T cells in vitro typically involve the use of engineered feeder cells, such as OP9‐DL4 and MS5‐DL1, which express Notch‐activating Delta‐like 4 (DL4) or Delta‐like 1 proteins (DL1), to enhance the T cell differentiation efficiency.^13^, ^14^ With the advancement of biomaterial technology, differentiation systems that target Notch signalling and rather than engineering feeder cells, are emerging. Coating the DL4‐Fc plate enhances T‐cell differentiation in vitro, and the differentiation efficiency is improved when VCAM‐1 is present.^15^ Furthermore, DL4‐μ beads, in conjunction with lymphopoietic cytokines, induce a sequential differentiation from CD34^+^ HSPCs to CD34^+^CD7^+^CD5^+^ pro‐T cells, ultimately leading to the formation of CD3^+^ T cells.^16^ The 3D differentiation systems, such as the PSC‐artificial thymic organoid (ATO) system or other thymic and lymph nodes‐like organs, provide efficient platforms for generating T cells from human PSCs.^17^, ^18^, ^19^

Indeed, despite significant advancements, the in vitro T cell differentiation from PSCs remains challenged by low differentiation efficiency, a complex process and the functional maturation of terminally differentiated cells.^16^, ^19^, ^20^ Therefore, there is an urgent need for a T cell differentiation platform that can efficiently produce large‐scale, functionally mature T cells from PSCs to meet the substantial clinical demand for T cells. The PSCs undergo multiple stages, including HSPCs, during in vitro differentiation, which mirrors the in vivo developmental process. Although the in vitro differentiation of HSPCs into T cells has achieved a relatively satisfactory efficiency, current protocols for differentiating PSCs into HSPCs still suffer from low differentiation efficiency and functional maturation of these HSPCs.^8^ This could represent a significant obstacle for acquiring T cells through in vitro differentiation of PSCs. Consequently, our goal is to develop a tool that can be easily used to identify both the HSPCs and T cells in differentiating cultures, thereby facilitating the optimization of HSPC and/or T cell differentiation procedures.

The transcription factor RUNX1 plays a pivotal role in promoting haematopoietic stem cell (HSC) specification by regulating the endothelial‐to‐haematopoietic transition (EHT)^21^, ^22^, ^23^, ^24^ Recent advancements in single‐cell RNA sequencing (scRNA‐seq) profiling on human haematopoietic tissues throughout gestation have revealed that RUNX1 is one of the most critical markers distinguishing HSCs from progenitors.^25^, ^26^ Another transcription factor, TCF7, also known as T‐cell factor 1 (TCF1), is predominantly expressed in lymphoid progenitor cells and T cells, but not in B cells and NK cells.^27^ TCF7 plays a crucial role in regulating T cell specification. The knockout of Tcf7 in the mouse haematopoietic progenitor stage results in the stem/progenitor‐cells being unable to develop into T cells.^28^

In this study, we engineered the GFP and mCherry into the RUNX1 and TCF7 loci of human iPSCs, respectively, creating the TCF7^mCherry^RUNX1^GFP^ double knock‐in iPSC line. The in vitro differentiation of TCF7^mCherry^RUNX1^GFP^ iPSCs into HSPCs and T cells demonstrated that GFP fluorescence closely correlates with CD34^+^ cells, and mCherry fluorescence closely correlates with CD3^+^ cells. This suggests that GFP and mCherry serve as effective reporters to indicate HSPC and T cell populations in differentiating cultures. Utilizing this TCF7^mCherry^RUNX1^GFP^ iPSC line, we optimized the in vitro T cell differentiation and determined that a mixture of OP9 stromal feeder cells overexpressing CXCL12 (OP9‐CXCL12) and DL4 (OP9‐DL4) at a 1:3 ratio (OP9‐C1D3) significantly improved CD8^+^ T cell differentiation from iPSCs.

## 2 RESULTS

### 2.1 Generating TCF7^mCherry^RUNX1^GFP^ double knock‐in reporter iPSC lines

The iPSC line Z1C1 was generated by Nuwacell Co., LTD. These cells exhibited the characteristic morphology of PSCs and maintained a normal karyotype (Figure S1A,B). They expressed pluripotency markers such as OCT4, SOX2 and NANOG. After being implanted into Severe combined immunodeficiency (SCID) mice, they formed teratomas (Figure S1C,D).

**FIGURE 1:**
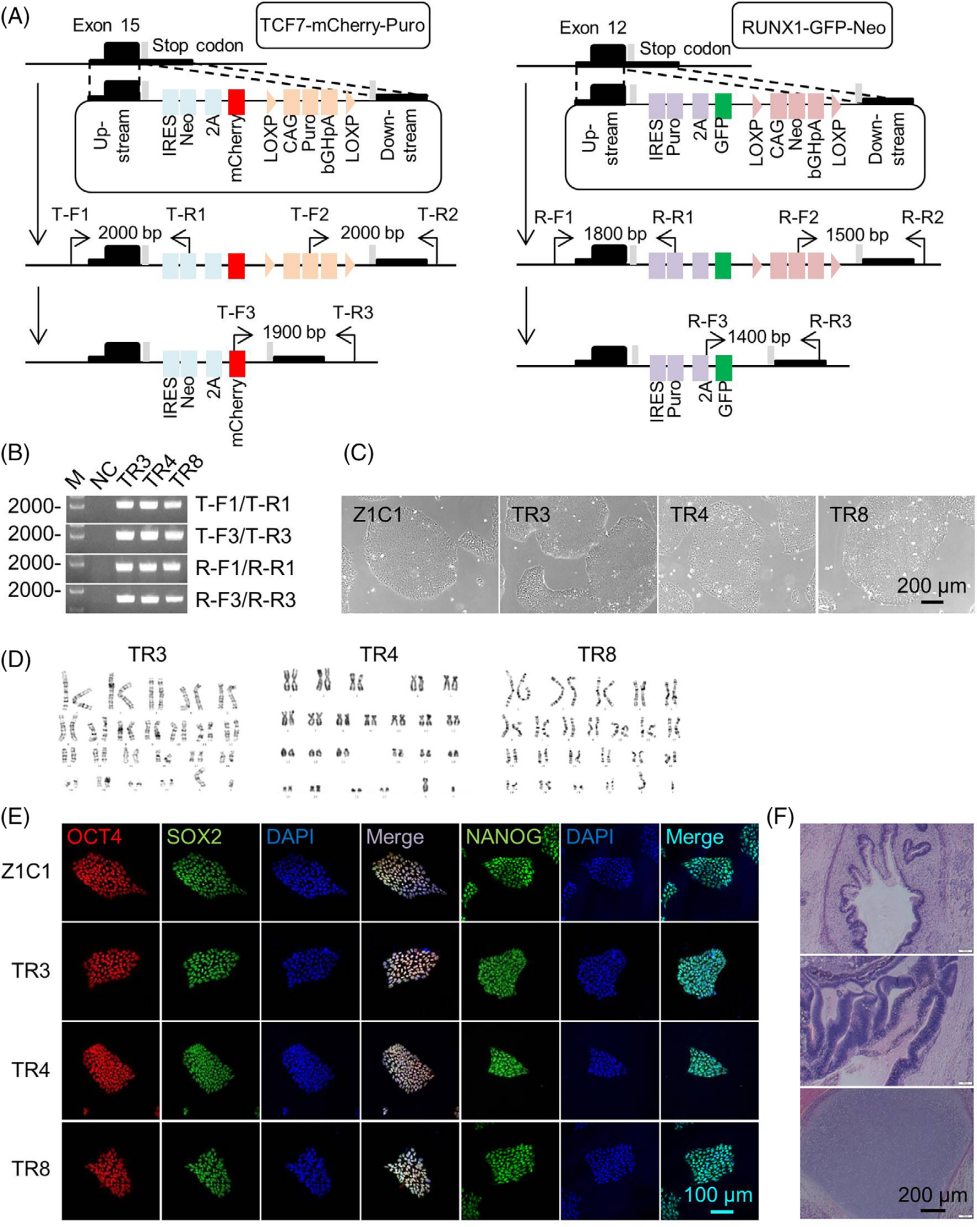
Generation of TCF7^mCherry^RUNX1^GFP^ double knock‐in iPSC (TR) lines. (A) A schematic diagram illustrates the construction of TCF7^mCherry^RUNX1^GFP^ double knock‐in iPSC (TR) lines. The left panel shows the donor vector TCF7‐mCherry‐Puro, including TCF7 upstream and downstream homologous arms, a neomycin resistant gene, the mCherry sequence, and a LOXP flanked CAG promoter‐driven puromycin resistant gene, and the primer locations for genotyping of TCF7 targeting. The right panel shows the donor vector RUNX1‐GFP‐Neo, consisting of RUNX1 upstream and downstream homologous arms, a puromycin resistant gene, the GFP sequence and a LOXP flanked CAG promoter‐driven neomycin resistant gene, and the primer locations for genotyping of RUNX1 targeting. (B) The TRs are identified by PCR. TR3, TR4 and TR8 represent the three TCF7^mCherry^RUNX1^GFP^ double knock‐in iPSC lines (M: DNA marker. NC: negative control. Amplification of Z1C1 genomic DNA was used as a negative control). (C) Representative phase contrast microscopy images of Z1C1, TR3, TR4 and TR8. (D) The TR3, TR4 and TR8 have normal karyotypes. (E) Immunofluorescence images display the expression of pluripotent genes OCT4, SOX2 and Nanog in Z1C1, TR3, TR4 and TR8. The cells were stained with anti‐OCT4 (red), anti‐SOX2 (green), anti‐NANOG (green) and DAPI (blue). (F) Representative H&E staining images of teratoma formed by TR. Top: endodermal tissues; middle: ectodermal tissues; bottom: mesodermal tissues.

To generate iPSCs with mCherry‐labelled TCF7 and GFP‐labelled RUNX1, the mCherry and GFP were designed to be integrated into the TCF7 and RUNX1 loci of iPSCs, respectively (Figure 1A). The TCF7‐mCherry‐Puro cassette, which includes a TCF7 upstream homologous sequence, the exogenous terminators, a neomycin, a mCherry, a LoxP flanked CAG promoter‐driven puromycin and a TCF7 downstream homologous sequence, was inserted immediately before the stop codon of the TCF7 locus (Figure 1A, left panel). The RUNX1‐GFP‐Neo cassette, consisting of a RUNX1 upstream homologous sequence, the exogenous terminators, a puromycin, a GFP, a LoxP flanked CAG promoter‐driven neomycin and a RUNX1 downstream homologous sequence, was inserted immediately after exon 12 of Runx1 locus (Figure 1A, right panel). The LoxP flanked CAG promoter‐driven puromycin and CAG promoter‐driven neomycin facilitate the selection of positive knock‐in colonies during screening. Meanwhile, the endogenous RUNX1 promoter‐driven neomycin and TCF7 promoter‐driven puromycin aid in the enrichment of HSPC and T cell populations during differentiation culturing (Figure 1A).

The iPSCs with mCherry‐labelled TCF7 and puromycin resistance (TCF7^mCherry‐Puro^) were initially obtained through CRISPR/Cas9 mediated genome editing by transducing the TCF7‐mCherry‐Puro cassette, gRNA1 and CRISPR/Cas9 into Z1C1 iPSCs (Figure S2A,B,D). Subsequently, the LoxP flanked puromycin cassette was deleted via CRE, resulting in the TCF^mCherry^ iPSC line (Figure S2A,D). The next step involved the insertion of the RUNX1‐GFP‐Neo cassette into the RUNX1 locus using gRNA2‐guided CRISPR/Cas9 genome editing, which led to the creation of the TCF7^mCherry^RUNX1^GFP‐Neo^ iPSC lines (Figure S2A,C,D). Lastly, the LoxP flanked neomycin was removed by CRE, generating the TCF7^mCherry^RUNX1^GFP^ iPSC (TR) lines TR3, TR4 and TR8 (Figure 1B and Figure S2A,D). These iPSC lines, TR3, TR4 and TR8, exhibited normal morphology and karyotypes (Figure 1C,D), and showed the same self‐renewal and proliferation abilities with Z1C1 (Figure S2E,F). TRs expressed pluripotency markers OCT4, SOX2 and Nanog at levels similar to the Z1C1 iPSC line and formed teratomas after being implanted into severe combined immunodeficiency (SCID) mice (Figure 1E,F).

### 2.2 The GFP fluorescence, indicating RUNX1 expression, serves as a reporter of HSPC commitments during TCF7^mCherry^RUNX1^GFP^ iPSC differentiation

**FIGURE 2:**
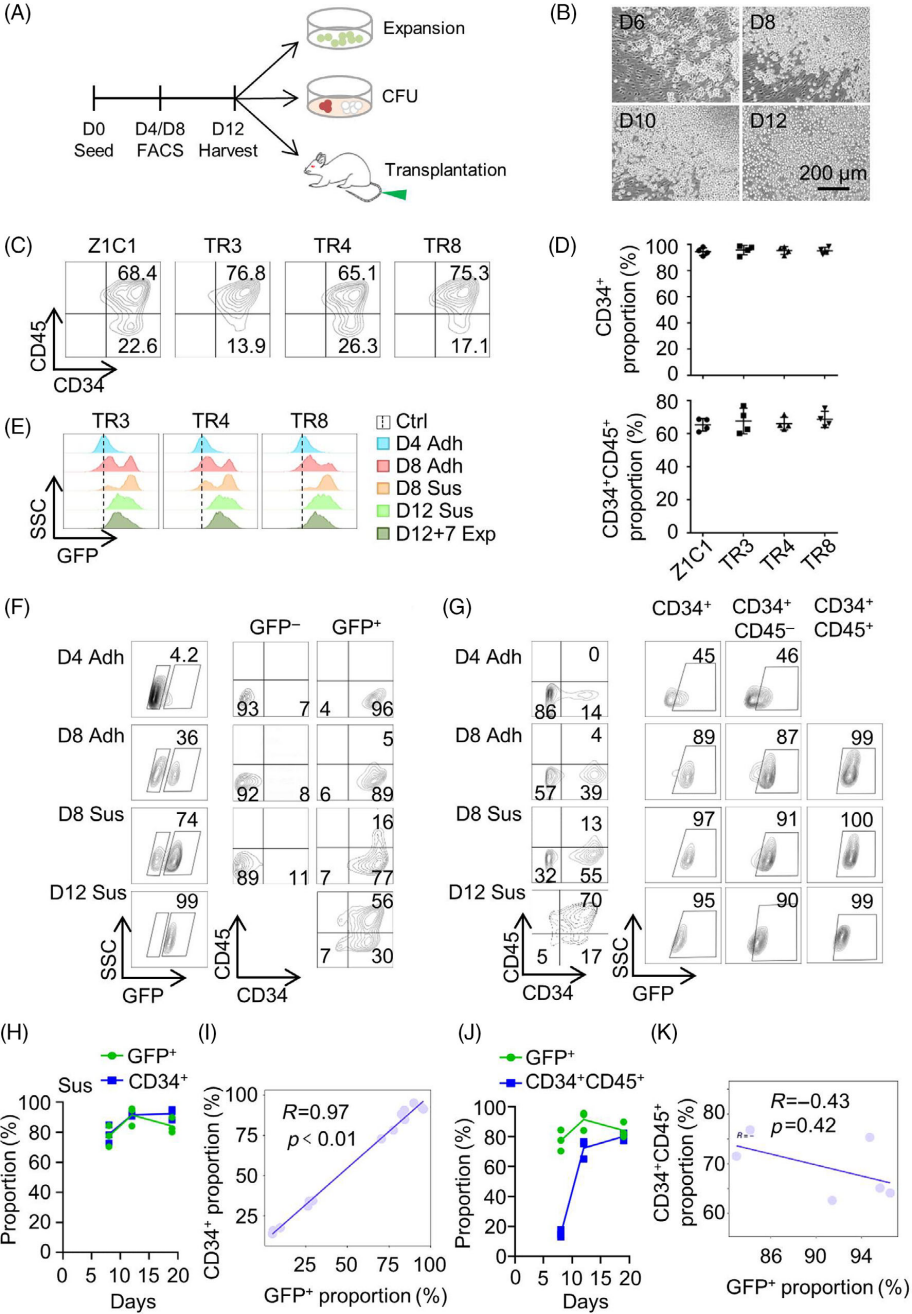
Correlations between the proportions of GFP^+^ cells and CD34^+^ cells differentiated from TRs. (A) A schematic graph illustrates the process for differentiation and functional evaluation of TR committed HSPCs. (B) Representative phase contrast microscopy images of differentiating TR4. (C) The expression of CD34 and CD45 in the differentiating HSPCs from Z1C1, TR3, TR4 and TR8 at day 12. (D) The proportions of CD34^+^ and CD34^+^CD45^+^ in the suspension populations of differentiating Z1C1, TR3, TR4 and TR8 (Data are shown as mean ± SD from four independent experiments). (E) The expression of GFP in HSPCs differentiated from Z1C1, TR3, TR4 and TR8 at indicated differentiating days. (F) The expression of CD34 and CD45 in the GFP^−^ and GFP^+^ cell populations. (G) The expression of GFP in the CD34^+^, CD34^+^CD45^−^ and CD34^+^CD45^+^ cell populations. (H) The proportions of GFP^+^ population and CD34^+^ population in suspension cells at indicated differentiating and expanding days (*n* = 3). (I) Scatterplots show the correlations between GFP^+^ proportion and CD34^+^ proportion (*n* =14). (J) The proportions of GFP^+^ population and CD34^+^CD45^+^ population in suspension cells at indicated differentiating and expanding days (*n* =3). (K) Scatterplots show the correlations between GFP^+^ proportions and CD34^+^CD45^+^ proportions (*n* =6). Ctrl, Z1C1; Adh, adherent cells; Sus, suspension cells; Exp, expansion. D, day; D12 + 7: the differentiating suspension cells at day 12, which then undergo a further 7 days of expansion.

To assess the feasibility of utilizing GFP fluorescence as a reporter of haematopoietic commitment during in vitro HSPC differentiation from TCF7^mCherry^RUNX1^GFP^ iPSCs, we firstly established a HSPC differentiation procedure using a commercial STEMdiff™ Haematopoietic Kit. The expansion capability of TCF7^mCherry^RUNX1^GFP^ iPSC‐committed HSPCs was evaluated by counting the changes in number over a 7‐day in vitro culture. The functionality of HSPCs was assessed by combing an in vitro colony‐forming unit assay and an in vivo immune deficient mouse transplantation assay (Figure 2A). During differentiation, haematopoietic‐like colonies emerged on day 6, and HSPC‐like cell layers formed from day 8 onwards (Figure 2B). Cells with a spherical morphology began to float in the supernatant of cultures from day 8 and continued until day 12 (Figure 2A,B). Both adherent and suspended globular cells were collected on day 12 and tested for the expression of CD34 and CD45 using a Fluorescence‐activated Cell Sorter (FACS) (Figure 2C,D). The CD34^+^ cells consistently accounted for more than 90% of suspended cells in the four differentiating cultures of TR3, TR4, TR8 and their parental iPSC line Z1C1, indicating that the engineering of mCherry into the TCF7 locus and GFP into the RUNX1 locus did not affect the haematopoietic differentiation ability of iPSCs. Notably, all four iPSC lines demonstrated high HSPC differentiation ability, as indicated by more than 65% of floating cells collected from the differentiating cultures of these cells being CD34^+^CD45^+^ (Figure 2C,D).

Next, we assessed the GFP fluorescence in both adherent cells and cells in suspension from the differentiating cultures. GFP fluorescence‐positive cells appeared in adherent cells as early as differentiation day 4, and their populations gradually increased throughout the differentiation process in both adherent and suspension cells (Figure 2E,F). By differentiation day 12, the vast majority of cells in suspension (~99%) were GFP positive (Figure 2E,F). The majority of GFP^+^ cells in each culture at any tested time points, whether they were adherent or in suspension, were CD34^+^ (Figure 2F). The CD34^+^CD45^+^ cells appeared in both adherent and suspension cells on differentiating day 8, and reached their highest proportion on differentiating day 12 (Figure 2F,G). The majority of GFP^−^ cells, whether they were adherent or in suspension, were CD34^−^CD45^−^, regardless of the differentiation time points (Figure 2F).

We then examined the expression of GFP in CD34^+^, CD34^+^CD45^−^ and CD34^+^CD45^+^ cell populations. At day 4 of differentiation, approximately 14% of adherent cells were CD34^+^, with no CD34^+^CD45^+^ cells detected, and less than half of CD34^+^ cells (~45%) were GFP^+^. At day 8 of differentiation, about 43% of adherent cells and 68% of suspension cells were CD34^+^, respectively, and the majority of these CD34^+^ cells were GFP^+^ (89% vs. 97%). Approximately 87% of adherent cells and 91% of suspension cells in the CD34^+^CD45^−^ populations were GFP^+^, while 99% of adherent cells and 100% of suspension cells in the CD34^+^CD45^+^ populations were GFP^+^. At day 12 of differentiation, around 87% of suspension cells were CD34^+^ cells, and 70% of these suspension cells were CD34^+^CD45^+^. Around 95% of CD34^+^ cells and 99% of CD34^+^CD45^+^ cells were GFP^+^ (Figure 2G).

We then conducted a statistical analysis to examine the correlations between the proportions of GFP^+^ cells and CD34^+^ cells, as well as the correlations between the proportions of GFP^+^ cells and CD34^+^CD45^+^ cells in the differentiating cultures. While the proportions of GFP^+^ cells and CD34^+^ cells showed a strong correlations, with a correlation efficiency of *R* = 0.97 (*p* < 0.01) (Figure 2H,I), the correlation between the proportions of GFP^+^ cells and CD34^+^CD45^+^ cells was lower (*R* = ‐0.43, *p* = 0.42) (Figure 2J,K). These findings suggest that GFP fluorescence, which indicates RUNX1 expression, could serve as a reporter for early haematopoietic commitment to CD34^+^ HSPCs, but not necessarily for defining specific CD34^+^CD45^+^ HSPC populations.

Following that, we tested the proliferation ability of GFP^+^ HSPC cells, which include both CD34^+^CD45^−^ and CD34^+^CD45^+^ cells. The GFP^+^ cells in suspension, collected from each differentiating culture of Z1C1, TR3, TR4 and TR8 at day 12, were propagated using the STEMdiff™ Haematopoietic Kit for 7 days. We counted the total cell numbers at both the beginning and end of the culturing period. The GFP^+^ HSPC cells from these four differentiating cultures demonstrated a very similar expansion potential (Figure S3A). The numbers of GFP^+^ HSPC cells from each of these differentiating cultures expanded more than five times compared to their initial seeding date (Figure S3A). Notably, the CD34^+^CD45^+^ cells were favourably enriched and the proportions of CD34^+^CD45^+^ cells significantly increased compared to their corresponding differentiating cultures at day 12 (Figure S3B,C).

### 2.3 GFP ^+^ HSPCs differentiated from TCF7^mCherry^RUNX1^GFP^ iPSCs demonstrate haematopoietic potency

We next employed the in vitro colony‐forming unit (CFU) assay and in vivo immune‐deficient mouse transplantation assay to evaluate the haematopoietic potency of GFP^+^ HSPCs differentiated from TCF7^mCherry^RUNX1^GFP^ iPSCs. We conducted standard CFU assays using suspension GFP^+^ cells from each of Z1C1, TR3, TR4 and TR8 differentiating cultures at day 12. The colonies of erythroid progenitor cells (CFU‐E and BFU‐E), granulocyte‐macrophage progenitor cells (CFU‐GM, CFU‐G, CFU‐M) and multipotential granulocyte, erythroid, macrophage and megakaryocyte progenitor cells (CFU‐GEMM) were identified based on their standard morphological criteria (Figure S3D). The numbers of burst‐forming unit‐erythroid (BFU‐E), colony forming unit‐erythroid (CFU‐E), colony forming unit‐granulocytes/macrophages (CFU‐G/M) and colony forming unit‐granulocyte/erythrocyte/macrophage /megakaryocyte (CFU‐GEMM) were quantified, and all of the four types of GFP^+^ HSPCs showed similar differentiation patterns in BFU‐E, CFU‐E, CFU‐GM and CFU‐GEMM (Figure S3D,E). These results indicate these cells possess similar abilities in proliferation and differentiation to erythroid and myeloid lineages.

To further demonstrate the haematopoietic potency of these in vitro differentiated GFP^+^ HSPCs, we transplanted the TR4 derived GFP^+^ HSPCs into immune‐deficient NOD/SCID mice. Four months post‐transplantation, we examined the haematopoietic lineage commitments in the bone marrow, peripheral blood and spleen of recipient mice. We detected human haematopoietic cells (hCD45^+^) in the bone marrow, blood and spleen of 4 out of the 8 transplanted NOD/SCID mice, and the CD34^+^ HSPCs were present at a varying frequencies in these transplanted mice (Figure S3F–H). We also detected B cells, NK cells, DC cells and monocytes in the bone marrow, peripheral blood and spleen of these transplanted mice at variable levels (Figure S3F–H). Interestingly, the human CD3^+^ cells detected in the bone marrow and spleen expressed the B cell marker CD19 (Figure S3F). These cells could potentially be a type of lymphoid progenitor of both B and T cells, as has been recently reported.^29^ The multi‐lineage commitments by TR4 derived GFP^+^ HSPCs in immune‐deficient NOD/SCID mice observed here suggest that the GFP^+^ HSPCs differentiated from iPSCs have the potential to differentiate into both lymphoid and myeloid lineages in vivo.

### 2.4 Optimizing in vitro T cell differentiation from HSPCs using mCherry fluorescence to indicate CD3 ^+^ T cells

**FIGURE 3:**
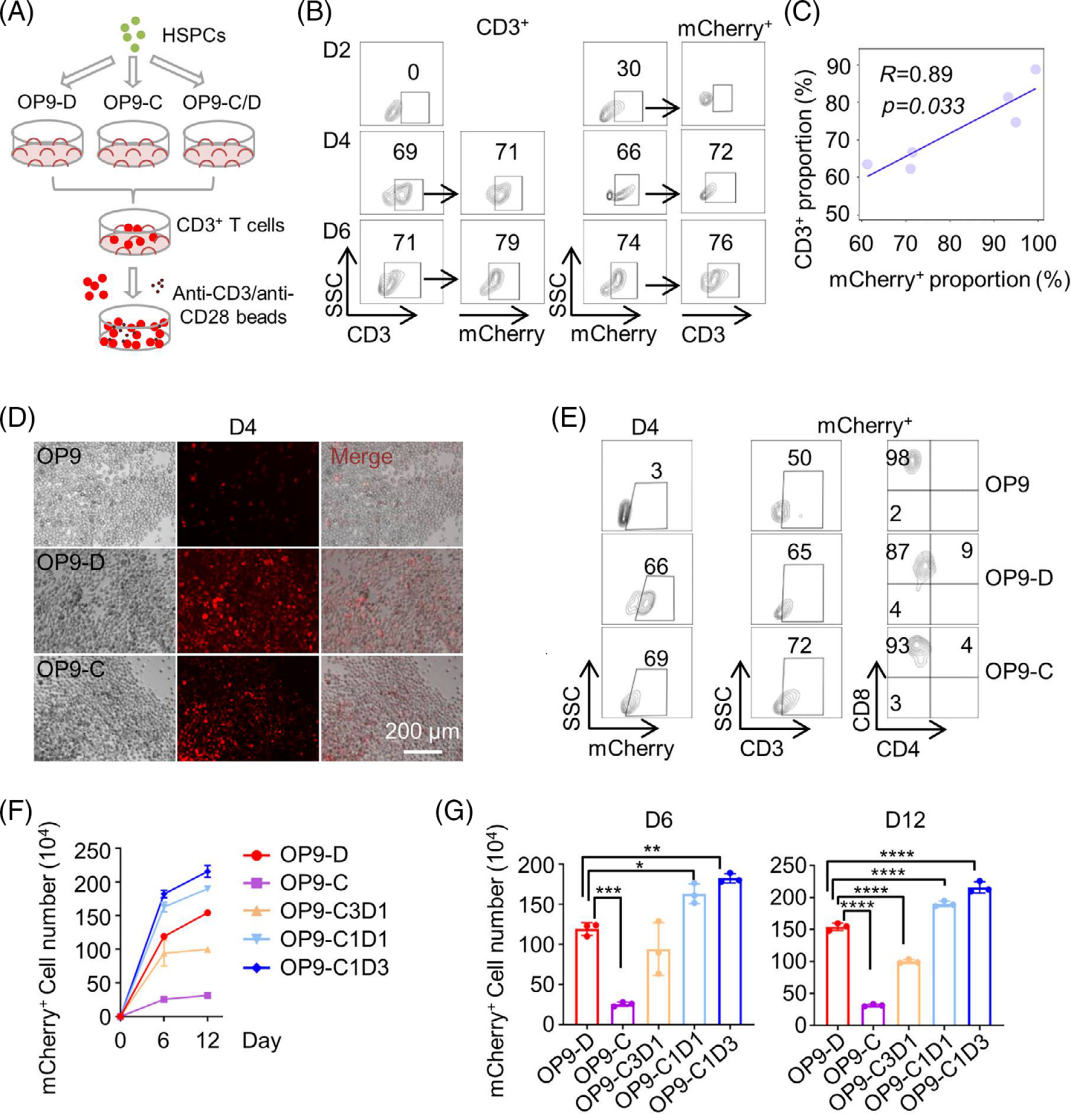
Optimizing in vitro T cell differentiation using TR. (A) A schematic graph is presented that outlines the optimization process for T cell differentiation from HSPCs. (B) The expression of mCherry in CD3^+^ cell populations (left 2 panels), and the expression of CD3 in mCherry^+^ cell populations (right 2 panels), at differentiating day 2, 4 and 6 on OP9‐DL4 feeder layers. (C) Scatterplots show the correlations between mCherry^+^ proportions and CD3^+^ proportions (*n* =6). (D) Representative mCherry fluorescence images of T cell differentiating cultures on the feeders of OP9, OP9‐DL4 and OP9‐CXCL12 at day 4. (E) The expression of CD3, CD4 and CD8 in the mCherry^+^ populations at differentiating day 4 on the feeders of OP9, OP9‐DL4 and OP9‐CXCL12. (F) The numbers of mCherry^+^ cells in the cultures on the indicated feeder layers at differentiating day 6 and 12. (G) Statistical analysis of the mCherry^+^ cells in F. Data were shown as mean ± SD from three independent experiments. Unpaired two‐tailed Student's *t*‐test, *p < 0.05, ** *p* < 0.01, *** *p* < 0.001, **** *p* < 0.0001. OP9‐D, OP9‐DL4; OP9‐C, OP9‐CXCL12; OP9‐C/D, OP9‐CXCL12 mixed with OP9‐DL4; OP9‐C3D1, OP9‐CXCL12 mixed with OP9‐DL4 at ratio of 3:1; OP9‐C1D1, OP9‐CXCL12 mixed with OP9‐DL4 at ratio of 1:1; OP9‐C1D3, OP9‐CXCL12 mixed with OP9‐DL4 at ratio of 1:3.

In our efforts to optimize in vitro T cell differentiation, we initially assessed the feasibility of using mCherry as a reporter to indicate T cell commitment during in vitro T differentiation from GFP^+^ HSPCs (Figure 3A). We first employed a traditional protocol, which involves the use of OP9 cells stably overexpressing DL4 (OP9‐DL4) as feeder cells, to differentiate TR4‐iPSC committed GFP^+^ HSPCs into CD3^+^ T cells (Figure 3A). CD3^+^ cells began to appear on differentiation day 4 and constituted approximately 70% of all cells in the differentiation culture on days 4 and 6 (Figure 3B). The mCherry^+^ cells appeared on differentiation day 2, and made up around 30% of all cells in the differentiation culture. On differentiation day 4 and day 6, mCherry^+^ cells accounted for about 51% and 74% of all differentiating cells, respectively (Figure 3B). Notably, the proportions of CD3^+^ cells and mCherry^+^ cells on differentiation day 4, 6 and 12 were closely correlated (*R* = 0.89, *p* = 0.033), supporting the use of mCherry fluorescence as a reporter to indicate T cell commitments during in vitro HSPC differentiation (Figure 3C and Figure S4A).

The role of the interaction between C–X–C motif chemokine ligand 12 (CXCL12), also known as pre‐B cell growth stimulating factor (PBSF)/stromal cell‐derived factor 1 (SDF‐1), and the G‐protein coupled receptor chemokine receptor 4 (CXCR4) in the survival, expansion and differentiation of human early thymocytes has been established, mirroring its counterpart in mice.^30^, ^31^ Consequently, we explored the potential of CXCL12 to enhance in vitro T cell differentiation. The TR4 iPSC committed HSPCs (TR4‐HSPCs) expressed CXCR4 (Figure S4B). We established and used OP9 stroma cells, which stably overexpress human CXCL12 (OP9‐CXCL12), as feeder cells to differentiate TR4‐HSPCs into T cells (Figure 3A). Interestingly, the use of OP9‐CXCL12 as feeder cells for T cell differentiation significantly increased the proportions of mCherry^+^ cells in the differentiating cultures, compared to using OP9 as feeder cells (Figure 3D–G). However, the numbers of mCherry^+^ cells in the differentiating cultures were significantly lower when using OP9‐CXCL12 only as feeder cells, compared to using OP9‐DL4 only as feeder cells, whether during the initial 6 days or on the 12th day of differentiation (Figure 3F,G and Figure S4C). This finding led us to investigate whether a combination of OP9‐DL4 and OP9‐CXCL12 as feeder cells could enhance T cell differentiation efficiency.

The efficiencies of T cell commitment, using OP9‐DL4, OP9‐CXCL12 or mixtures of OP9‐CXCL12 and OP9‐DL4 at ratios of 1:3 (OP9‐C1D3), 1:1 (OP9‐C1D1) and 3:1 (OP9‐C3D1) as feeder cells, were evaluated by quantifying the proportion of mCherry^+^ cells in differentiating cultures on day 6 and day 12. Interestingly, the use of a mixture of OP9‐CXCL12 and OP9‐DL4 at a ratio of 1:3 as feeder cells achieved significantly enhanced mCherry^+^ cell commitment efficiency, compared to using only OP9‐DL4 as feeder cells (Figure 3F,G). Similar results were obtained when CD3^+^ was used to represent T cells instead of mCherry^+^ (Figure S4D,E). It's worth noting that the majority of CD3^+^ cells differentiated in our system were CD8^+^ cells, with no CD4^+^ cells observed (Figure S4F,G).

### 2.5 Optimizing in vitro HSPC/T cell differentiation using Puro and Neo

Next, we tested the Puro effects on production of HSPCs. Flow cytometry analysis results indicated that a 3‐day Puro treatment resulted in approximately 30% of CD34^+^CD45^+^ cells on the 8th day of HSPCs differentiation. This contrasted with the counterpart, which only had about 20% of CD34^+^CD45^+^ cells present at the same time (Figure S5A). By the 12th day, a higher number of CD34^+^CD45^−^ cells and a lower number of CD34^−^ cells were collected in suspension when Puro was administered (Figures S5B–F).

We also tested the Neo effects on T cell production. Flow cytometry analysis results indicated that after a 6‐day period of adding Neo, the proportion of CD8 cells decreased with unknown reason (Figure S5G). However, in contrast, after a 6‐day treatment with Neo, we were able to obtain a higher number of CD3^+^ cells compared to the control group (Figure S5H).

### 2.6 Chimeric antigen receptor T (CAR‐T) cells differentiated from TR4 iPSC containing CAR (CAR‐TR4) demonstrated effective cytotoxicity in vitro

**FIGURE 4:**
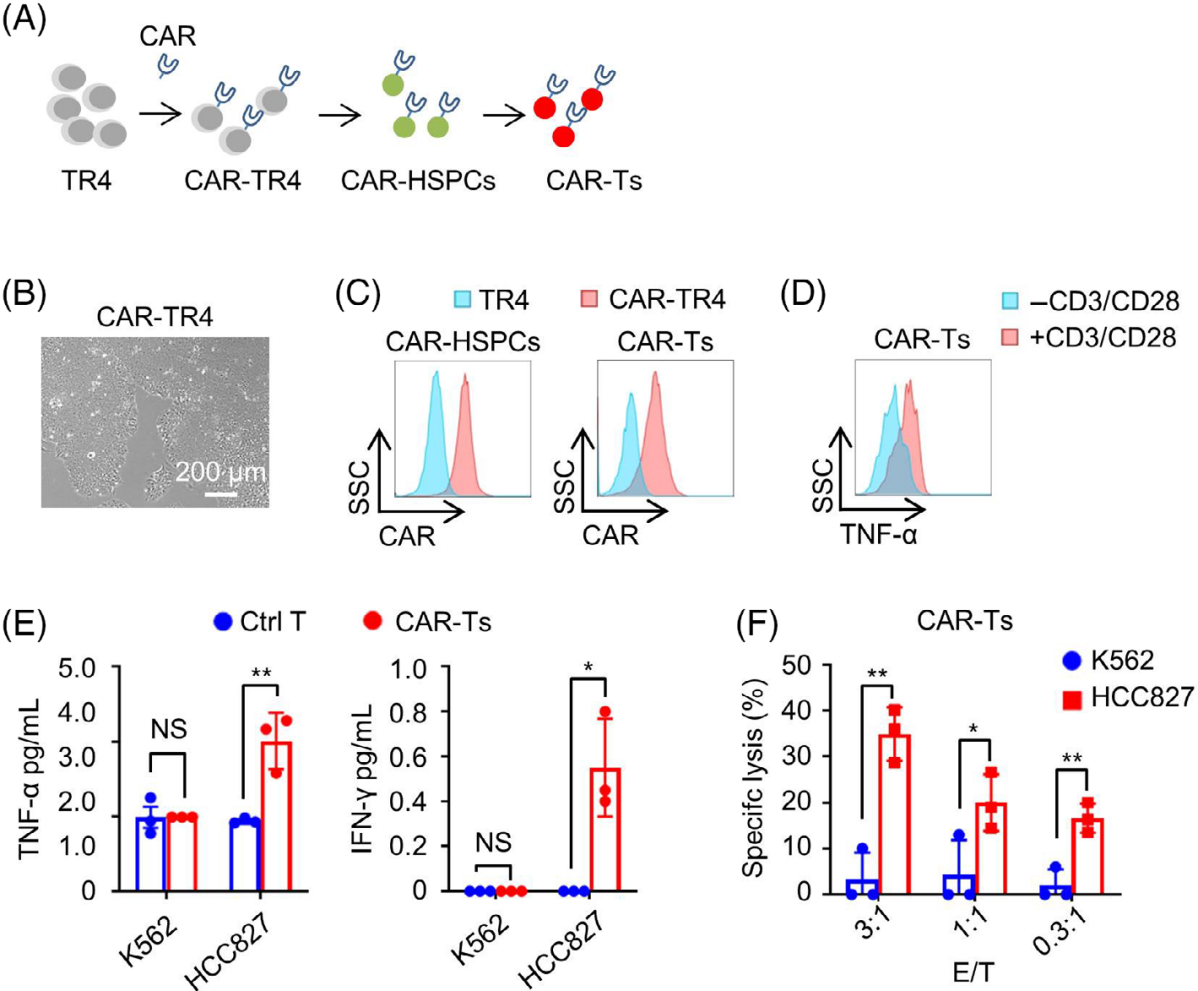
CAR‐T cells differentiated from TR show effective cytotoxicity in vitro. (A) A schematic diagram is presented that shows the generation of CAR‐T cells from TR4 iPSCs. (B) A representative phase contrast microscopy image is shown of TR4 iPSCs that have been transduced with CAR, creating CAR‐TR4. (C) CAR is expressed in both HSPCs derived from CAR‐TR4 iPSCs (CAR‐HSPCs) and T cells derived from CAR‐HSPCs (CAR‐Ts). TR4 iPSCs, which do not express CAR, were used as a negative control. (D) The TNF‐α is expressed in TR4 derived CAR‐Ts upon anti‐CD3/anti‐CD28 stimulation. (E) The CAR‐T cells derived from CAR‐TR4 secreted both TNF‐α and IFN‐γ when co‐cultured with EGFR‐positive HCC827 cells but not with EGFR‐negative K562 cells. Ctrl T refers to T cells differentiated from TR4 iPSCs. Data are shown as the mean ± SD of three independent experiments. Unpaired two‐tailed Student's *t*‐test. NS, not significant, *p < 0.05, **p < 0.01. (F) The TR4‐CAR‐T cells lyse HCC827 but not K562 in vitro. Data are shown as the mean ± SD from three independent experiments. Unpaired two‐tailed Student's *t*‐test. NS, not significant, *p < 0.05, **p < 0.01.

We proceeded to test the feasibility of engineering CAR into iPSCs and differentiating these cells into ready‐to‐use CAR‐T cells, which could be used in CAR‐T mediated cancer immunotherapy. We introduced a previously reported CAR construct that targets EGFR into TR4 iPSCs, thereby generating CAR‐TR4 iPSCs (Figure 4A,B).^32^ These cells were then differentiated into GFP^+^ HSPCs (CAR‐HSPCs) and mCherry^+^ CD8^+^ T cells (TR4‐CAR‐T) using the aforementioned optimized differentiation settings. Both T cells and CAR‐T cells differentiated from TR are TCRαβ^+^ with limited TCRγδ^+^ (Figure S6A). The CAR‐HSPCs and TR4‐CAR‐T cells that were differentiated from CAR‐TR4 iPSCs consistently expressed CARs (Figure 4C). When stimulated with anti‐CD3 and anti‐CD28 antibodies, the TR4‐CAR‐T cells expressed TNF‐α in vitro (Figure 4D).

We proceeded to co‐culture TR4‐CAR‐T cells with human lung cancer cell lines K562 (which does not express EGFR) and HCC827 (which does express EGFR). Unlike control T cells, which were not activated by either K562 or HCC827 cancer cells, TR4‐CAR‐T cells were activated to secrete TNF‐α and IFN‐γ by HCC827 but not by K562 (Figure 4E). In line with this, cell co‐culture assays demonstrated that HCC827 cells, but not K562 cells, were efficiently lysed by TR4‐CAR‐T cells (Figure 4F).

## 3 DISCUSSION

In vitro T cell differentiation from PSCs offers a potentially unlimited source of T cells for cancer immunotherapy. However, the low efficiency of PSC differentiation into functional mature T cells currently limits their widespread applications. Traditional protocols for identifying factors that facilitate T cell differentiation from PSCs involve detecting a range of HSPC and T cell markers, a process that is laborious and difficult to scale up. In this study, we developed a double knock‐in iPSC tool by integrating mCherry into the TCF7 locus and GFP into the RUNX1 locus, which simplifies the optimization of in vitro T cell differentiation. The expression of GFP fluorescence accurately represents CD34^+^ HSPC commitments, while mCherry fluorescence indicates T cell commitments during in vitro differentiation culturing. This dual fluorescence reporter system enables a fast, efficient and scalable screening process. As a proof‐of‐concept, we quickly identified a feeder recipe, consisting of a 1:3 ratio of CXCL12‐expressing OP9 stroma cells and DL4‐expressing OP9 cells, which significantly enhanced T cell differentiation. We also engineered a CAR targeting EGFR into iPSCs, creating CAR‐TR4 iPSCs for the differentiation of ready‐to‐use CAR‐T cells. The CAR‐T cells differentiated from these CAR‐TR4 iPSCs using OP9‐C1D3 as feeder cells demonstrated effective cytotoxicity against cancer cells, validating the functionality of these iPSC‐derived T cells.

The transcription factor RUNX1 is crucial in regulating the expression of genes essential for definitive haematopoiesis and is required for the transition from endothelial to haematopoietic cells in mice.^21^, ^24^, ^33^ Recently, RUNX1 has been identified as one of the signature genes that distinguish human haematopoietic stem cells (HSCs) from haemogenic lineage‐restricted progenitors at all developmental stages.^25^, ^26^ RUNX1 regulates CD34 expression by directly binding to its downstream regulatory element in human long‐term HSCs.^34^ As anticipated, the expression of GFP, which indicates RUNX1 expression, is closely correlated with CD34 expression in our system. This supports the use of GFP fluorescence as an indicator of CD34^+^ HSPC commitment. The differentiation of HSPCs into T cells requires the activation of multiple transcription factors (TFs). TCF7 is highly expressed in early thymic progenitors and its expression is upregulated by Notch signalling. TCF7 promotes the expression of T‐lineage genes, including T cell specific TFs GATA3 and BCL11B, and the TCR component CD3.^12^, ^35^, ^36^, ^37^, ^38^ The close correlation between the expressions of CD3 and TCF7 during T cell differentiation from PSCs allows us to use mCherry, which indicates TCF7 expression, as a reporter for T cell commitment.

CXCL12 is a member of the intercrine family, specifically an alpha chemokine derived from stromal cells. Initially, it was identified as a factor that stimulates pre‐B‐cell development.^39^ Subsequent research highlighted its vital role in maintaining the haematopoietic stem cell (HSC) pool in murine bone marrow stromal cell niches through interaction with CXCR4.^40^ Furthermore, the CXCL12/CXCR4 signalling pathway plays a key role in T cell development. It not only draws T cell progenitors to the thymus tissue regions but also fosters the survival and expansion of T cell precursors in mice^30^, ^41^, ^42^, ^43^ and zebrafish.^44^ In humans, early T and B lymphoid precursors exhibit high CXCR4 expression,^45^, ^46^ and the CXCL12‐CXCR4 signalling pathway is crucial for the survival, proliferation and differentiation of intrathymic T cell precursors.^31^ Interestingly, our experiments revealed that T cell commitment remained unaffected when we introduced CXCL12 proteins during the in vitro T cell differentiation process (data not shown). This observation is consistent with previous research, which found that the direct addition of CXCL12 proteins to T cell differentiating cultures did not enhance mouse T cell differentiation.^47^ However, when we combined OP9‐CXCL12 cells with OP9‐DL4 feeder cells during in vitro human T cell differentiation, we noticed a significant increase in the production of human T cells. This implies that the role of CXCL12/CXCR4 signalling in promoting T cell differentiation from PSCs might be dependent on cell–cell contact. Early studies have shown that the CXCL12/CXCR4 axis is critical for stromal cell contact‐mediated human early T lymphoid precursor (ETP) commitment from HSPCs, but not necessary for stromal cell contact‐independent ETP generation.^48^ These studies collectively suggest that the increased in vitro T cell differentiation efficiency observed here, achieved by incorporating OP9‐CXCL12 with OP9‐DL4, might be due to enhanced ETP commitments from HSPCs via a stromal cell contact‐dependent CXCL12/CXCR4 signalling.

Our refined differentiation system is capable of obtaining HSPCs and T cells with similar phenotypes in a shorter timeframe compared to other existing systems (Figure S6B).^8^, ^49^ Additionally, the majority of the T cells we obtained were TCRαβ^+^ cells, with limited number being TCRγδ^+^ cells (Figure S6A). These findings align with other T cell differentiation studies.^19^, ^20^

In conclusion, we have developed a human iPSC tool for optimizing in vitro T cell differentiation procedures. This tool uses the expression of GFP and mCherry fluorescences to directly report the differentiation of HSPCs and T cells. This will enable the development of protocols to quickly and efficiently screen factors that promote HSPC and/or T cell differentiation from PSCs. Using this tool, we have defined a more efficient T cell differentiation protocol that incorporates OP9‐CXCL12 feeder cells with traditional OP9‐DL4 feeder cells. We anticipate that this tool will endow a fast, efficient and scalable optimization procedure for in vitro T cell differentiation, serving T cell based immune therapies, as well as aid in the study of the developmental process of HSPCs and T cells.

## 4 MATERIALS AND METHODS

### 4.1 Maintenance of iPSCs

The human iPSC line Z1C1 was generated by Nuwacell Co., Ltd. These cells were grown in a feeder‐free system using human ESC/iPSC ncEpic Basal Medium (nuwacell), supplemented with 1× ncEpic Supplement (nuwacell) and maintained at 37°C with 5% CO_2_. Additionally, they were treated with 10 mM ROCK inhibitor Blebbistatin (nuwacell) during passage. The cells were subcultured every 4 days.

### 4.2 Nucleofection

The iPSCs were dissociated into individual cells through Solase (nuwacell) treatments and then centrifuged at 160*g* for 3 min. The iPSCs (8 × 10^5^) were then electroporated with the indicated constructs using a Human Stem Cell Nucleofector Kit 2 (Lonza) and Lonza Amaxa Nucleofector 2b (program A‐023) (Lonza), following the instructions in the user manual. After transfection, the cells were placed in six‐well plates (NUNC) and sub‐cultured for the intended purpose using ncEpic Medium supplemented with 10 mM Blebbistatin.

### 4.3 Generation of RUNX1^GFP^TCF7^mCherry^

Vectors containing sgRNA1 targeting TCF7, TCF7‐mCherry‐Puro constructs, and CRISPR/Cas9 were introduced into Z1C1 simultaneously using nucleofection. Following 3 days of treatment with puromycin at a concentration of 1 μg/mL, surviving cells underwent single‐cell sub‐cloning. The puromycin‐resistant colonies were then validated through PCR amplification, sequencing and southern blot. The resulting iPSCs were named TCF7^mCherry‐Puro^. The puromycin cassette was subsequently removed by transfecting a construct containing the cDNA of the CRE enzyme using nucleofection, resulting in TCF7^mCherry^ iPSCs.

Vectors containing sgRNA2 targeting RUNX1, RUNX1‐GFP‐Neo constructs and CRISPR/Cas9 were introduced into TCF7^mCherry^ cells using nucleofection. After a week of neomycin treatments at a concentration of 100 μg/mL, surviving cells were also subjected to single‐cell sub‐cloning. The positive colonies were confirmed through PCR amplification, sequencing and southern blot. These colonies, named TCF7^mCherry^RUNX1^GFP‐Neo^, underwent Cre‐mediated removal of neomycin, resulting in TCF7^mCherry^RUNX1^GFP^.

### 4.4 Genotyping

Genomic DNAs from the relevant cell lines were extracted using isopropanol precipitation. The intended fragments were then amplified using KOD DNA polymerase, a product of TOYOBO LIFE SCIENCE. The process involved the use of specific primers: TCF7: T‐F1: ACTGGATAAAATGGGTAGG, T‐R1: GCAATATGGTGGAAAATAAC; T‐F2: GGGGAGGATTGGGAAGACA, T‐R2: ACCAGTGGCTAAAGTAGGA; T‐F3: CTACGACGCTGAGGTCAA, T‐R3: TAAGGGGCTTCCTCTGGT. RUNX1: R‐F1: GCAGTCCCTATACTGACCA, R‐R1: GTCGCTACAGACGTTGTTT; R‐F2: GCTTCCTCGTGCTTTACGGTATC, R‐R2: CTTGGAGTGACCCTCAGTGTC; R‐F3: CGCAACCTCCCCTTCTACG. R‐R3: CCCCAACCAAAATTCCACA. The PCR products were verified by sequencing (GENEWIZ, China).

### 4.5 Southern blotting

The Southern blotting was carried out as previously described.^50^ The DNA sequence of the puromycin gene was amplified by PCR and used as probes. The primers were used for this process as follows: F: ATGACCGAGTACAAGCCCAC; R: GGCACCGGGCTTGCGGGTC.

### 4.6 Immunofluorescence

The indicated iPSCs were placed in 12‐well plates from NUNC on Matrigel‐coated cover glasses from Corning for a period of 1–2 days prior to staining. The cells were then fixed with a 4% polyformaldehyde solution for 10 min, permeabilized with a 0.5% TritonX‐100 solution for 15 min and blocked with BSA for 30 min at room temperature. The cells were then incubated with primary antibodies, which included Anti‐Oct3/4 from SANTA CRUZ BIOTECHNOLOGY, Anti‐Sox2 from Merck Millipore and Anti‐Nanog from Abcam, at 37°C for 1 h. They were then labelled with secondary antibodies, specifically Goat anti‐Mouse IgG (H + L) Highly Cross‐Adsorbed Secondary Antibody and Goat anti‐Rabbit IgG (H + L) Highly Cross‐Adsorbed Secondary Antibody, both from Invitrogen, for 1 h at room temperature. The cell nuclei were counterstained with DAPI. Images of the cells were captured using a Zeiss LSM880 Fast Ariyscan confocal microscope.

### 4.7 Flow cytometry

For the staining of surface marker proteins, a total of 1 million cells were first washed with DPBS and then suspended in 100 μL DPBS with Fc for 10 min. After this, they were incubated with the indicated antibodies for 20 min at a temperature of 4°C. The antibodies used were fluorescently‐labelled and included anti‐CD34‐PE, anti‐CD45‐APC, anti‐CD3‐FITC, anti‐CD4‐Brilliant Violet (BV) 421, anti‐CD8‐APC/Cy7, anti‐HA.11‐tag‐APC, anti‐CD3‐BV 510, anti‐CD19‐PerCP/Cy7, anti‐CD94‐PerCP/Cy5.5, anti‐CD11c‐PE/Cy7, anti‐NKP46‐PE/Cy7 and anti‐CD11b‐PE, all of which were obtained from Biolegend.

For intracellular staining, the cells were fixed and permeabilized using the Intracellular Fixation & Permeabilization Buffer Set from eBioscience, and then stained with anti‐TNF‐α and anti‐IFN‐γ antibodies, also from Biolegend, in perm buffer.

All cells that were stained with the corresponding antibodies were washed twice with DPBS, re‐suspended in 300 μL DPBS, and then subjected to a FACS analysis using a BD machine. The data from this analysis were then interpreted using FlowJo software.

### 4.8 HSPC differentiation

The STEMdiff™ Haematopoietic Kit from STEMCELL TECHNOLOGIES was utilized to derive HSPCs, following the instructions provided in the operation manual. Briefly, the iPSC colonies were treated with hPSC dissociation buffer for 7 min and then transferred to a matrigel‐coated plate at a density of 20–80 cell masses per well in a 12‐well plate. The culture medium was changed according to the instructions. The differentiating cells were analysed at specified time points. It was observed that the number of adherent blood island‐like colonies decreased around 12 days after differentiation.

### 4.9 HSPC expansion

The HSPCs derived from iPSCs were collected on the 12th day of differentiation and re‐suspended in Stemspan SFEMII medium, which was supplemented with 1× Stemspan CD34^+^ Supplement. These cells were then placed in a six‐well plate and incubated at a temperature of 37°C in an environment with 5% CO_2_ for a period of 7 days. The number of cells was quantified at the specified dates.

### 4.10 Colony‐forming unit (CFU) assays

The HSPCs in suspension at the 12th day of differentiation were used for CFU (Colony Forming Unit) assays. Approximately 10,000 suspension HSPCs were combined with 3 mL of MethoCult™ H4435 Enriched from STEMCELL Technologies, and then equally distributed into two wells of a six‐well plate from NUNC. These cells were incubated at 37°C with 5% CO_2_ for a week. The burst‐forming unit‐erythroid (BFU‐E), colony forming unit‐erythroid (CFU‐E), colony forming unit‐granulocytes/macrophages (CFU‐G/M) and colony forming unit‐granulocyte/erythrocyte/macrophages/megakaryocyte (CFU‐GEMM) were identified based on their typical morphologies, and their numbers were quantified.

### 4.11 HSPC engraftment

The NOD.CB17‐Prkdc^scid^Il2rg^tm1^/Bcgen (NOD/SCID) immune‐deficient mice, aged 7 weeks, were procured from Biocytogen. These mice underwent half‐lethal irradiation and were then injected with 10^5^ GFP^+^ HSPCs in 200 μL DPBS via the tail vein. Mice that were administered the same volume of DPBS served as negative controls. The peripheral blood of these mice was examined at the 4th, 8th and 12th weeks post‐transplantation. At the 16th week post‐transplantation, cells from the bone marrow, blood, spleen and thymus of these mice were analysed for haematopoietic lineage commitments using FACS.

### 4.12 T cell differentiation

Each of the feeder cells, namely OP9‐DL4, OP9‐CXCL12 or mixtures of OP9‐DL4 and OP9‐CXCL12 at ratios of 1:3, 1:1 and 3:1, were seeded into six‐well plates coated with 0.1% gelatin 1–2 days prior to use, aiming for a 70%–80% confluence monolayer. On the HSPC seeding date, approximately 15,000 HSPCs were plated on the indicated feeder monolayers in 2 mL of OP9 medium (α‐MEM with 20% FBS, 2 mM GlutaMAX, 100 U/mL penicillin–streptomycin) supplemented with SCF (10 ng/mL), IL‐7 (5 ng/mL) and Flt3L (10 ng/mL). The differentiating cells were collected and transferred to new plates with feeder monolayers every 6 days.

### 4.13 T cell stimulation

The specified T cells, cultured in a medium composed of X‐VIVO 15, 5% FBS, 1 mM sodium pyruvate, 2 mM GlutaMAX, IL‐7 (10 ng/mL) and IL‐15 (10 ng/mL), were treated with microbeads containing anti‐CD3/anti‐CD28 antibodies as previously reported in Jin, 2021. The cell numbers were quantified every 3 days.

### 4.14 Cytokine secretion

CAR‐T cells were co‐cultured with the specified target tumour cells at an effector‐to‐target (E/T) ratio of 3:1, which equates to 1.5 × 10^4^ CAR‐T cells versus 5 × 10^3^ tumour cells (either K562 or HCC827), for a duration of 24 h. Following this, the supernatants from these co‐cultures were collected and tested for the presence of specific cytokines (TNF‐α, IFN‐γ) using an enzyme‐linked immunosorbent assay (ELISA) (MULTI SCIENCE), as per the manufacturer's guidelines. The data were gathered using the EPOCH2 (BioTek). The concentrations of the cytokines were quantified using a standard curve.

### 4.15 Cytotoxicity assay

The K562 and HCC827 cells were transduced with a construct that expresses a green fluorescent protein‐firefly luciferase fusion protein (GFP/LUC). These cells were then co‐cultured with CAR‐T cells at varying quantities (1.5 × 10^4^, 5 × 10^3^ and 1.5 × 10^3^) for a period of 24 h. After this incubation period, D‐luciferin (YEASEN) at a concentration of 150 μg/mL was added to these co‐cultures. The intensity of the luciferance was then detected after 2 min.

## AUTHOR CONTRIBUTIONS

TZ and YZ designed the experiments; YZ, HX, CC, WC and ST performed the experiments and analysed the data; YZ, JC and TZ wrote the manuscript; TZ supervised the experiments.

## CONFLICT OF INTEREST STATEMENT

The authors declare no competing financial interests.

## Supporting information


**Data S1:** Supporting Information.

## Data Availability

The raw data supporting the conclusions of this article will be made available by the authors, without undue reservation.
