# Supplementary material for: Optimizing in vitro T cell differentiation by using induced pluripotent stem cells with GFP‐RUNX1 and mCherry‐TCF7 labelling
Source: Cell Prolif. 2024 Jun 10;57(10):e13661. doi: 10.1111/cpr.13661 (PMC11471423; doi:10.1111/cpr.13661)
Supplement: Supplementary file 1 — Data S1: Supporting Information. [file CPR-57-e13661-s001.docx]

##
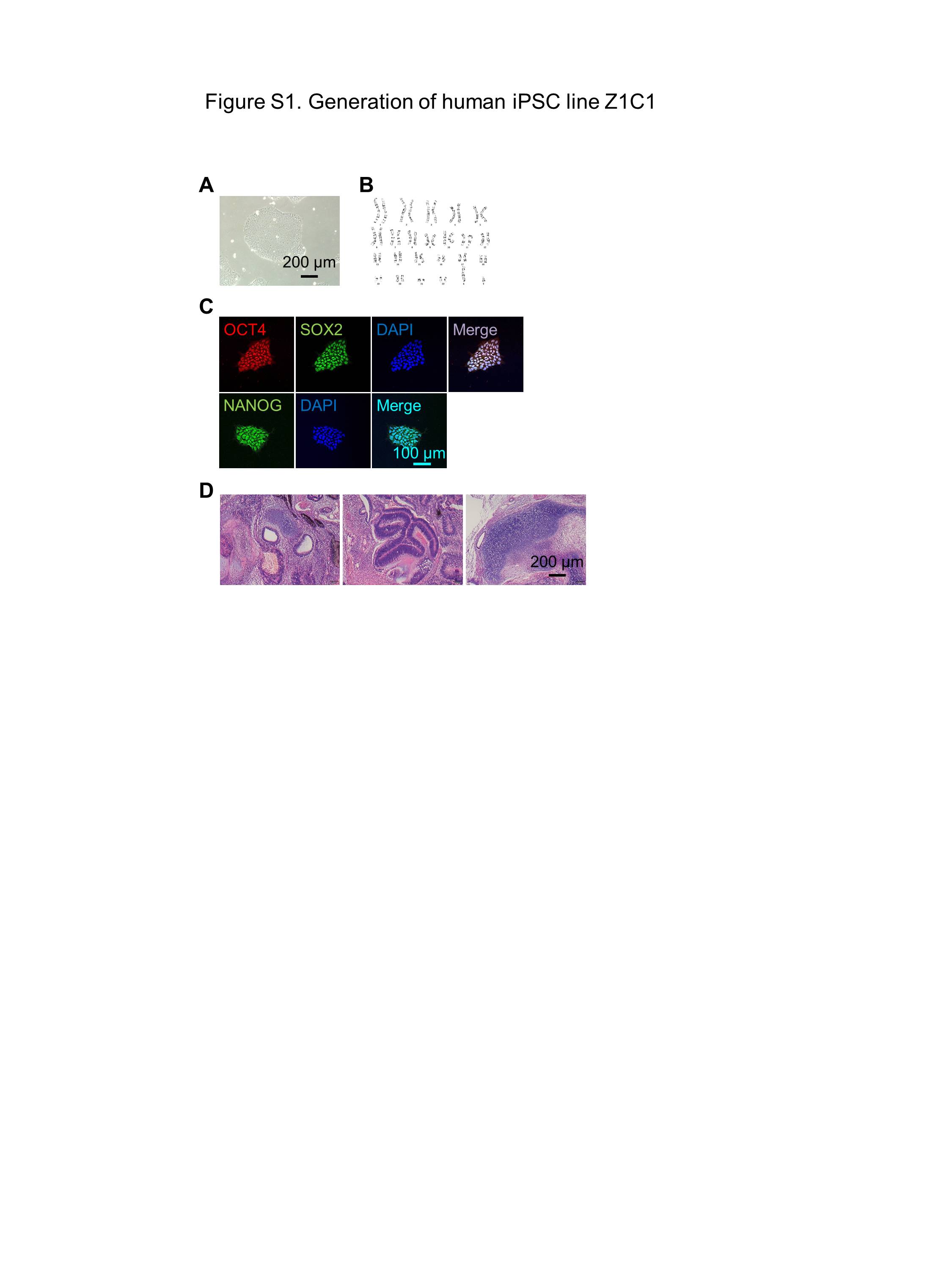


## Figure S1. Generation of human iPSC line Z1C1

# **(A)** A representative phase contrast microscopy image shows Z1C1 iPSCs. **(B)** Z1C1 iPSCs possess a normal karyotype. **(C)** Immunofluorescence images show the expression of pluripotent genes OCT4, SOX2, and Nanog in Z1C1. The cells are stained with anti-OCT4 (red), anti-SOX2 (green), anti-NANOG (green) and DAPI (blue). **(D)** Representative H&E staining images of teratoma formed by Z1C1. Left: endodermal tissues; center: ectodermal tissues; right: mesodermal tissues.

#
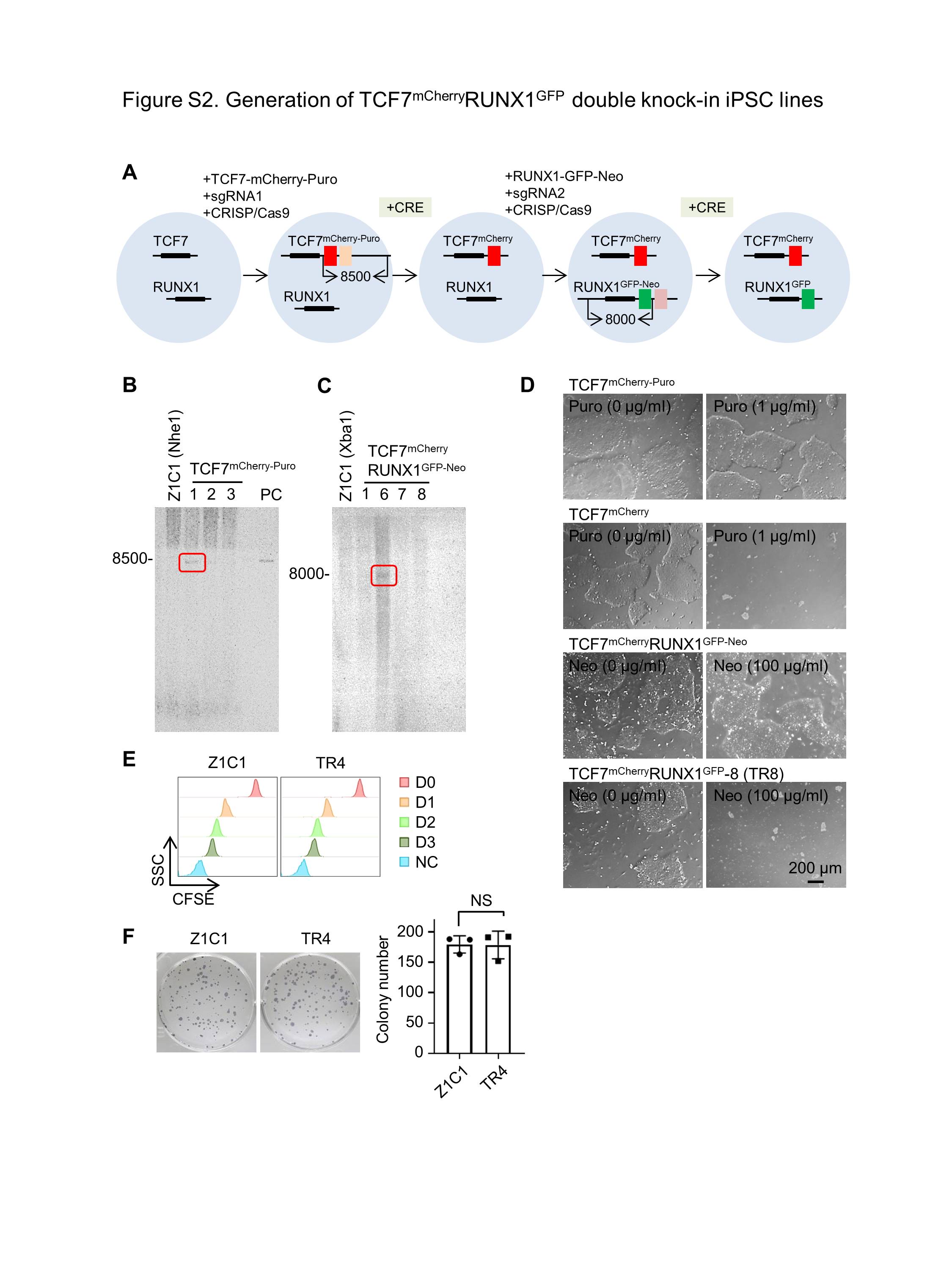


# **Figure S2. Generation of TCF7^mCherry^RUNX1^GFP^ double knock-in iPSC lines**

# **(A)** A schematic diagram is presented that shows the construction process for TCF7^mCherry^RUNX1^GFP^ double knock-in iPSC lines. gRNA1 and gRNA2 are guidance RNAs for TCF7 and RUNX1 targeting, respectively. **(B)** Southern blot analyses show the detecting of TCF7^mCherry-Puro^ sub-clones by using puromycin as probes. The genomic DNA of Z1C1, digested with Nhe1 restrictive endonuclease, serves as a negative control. The donor vector serves as a positive control for blotting. **(C)** Southern blot analyses show the detection of TCF7^mCherry^RUNX1^GFP-Neo^ sub-clones by using puromycin as probes. The genomic DNA of Z1C1, digested with Xba1 restrictive endonuclease, serves as a negative control. **(D)** Drug resistance testing for indicated intermediate and terminal iPSC lines. Puro, puromycin; Neo, neomycin. **(E)** CFSE analysis compares the proliferation abilities of Z1C1 and TR4. D0/1/2/3/4, day 0/1/2/3/4. **(F)** TR4 demonstrates the same self-renewal capacity as Z1C1. Left, representative images of alkaline phosphatase staining of colonies formed by Z1C1 and TR4. Right, statistical analysis of the number of alkaline phosphatase-positive colonies. Data are shown as the mean ± SD from three independent experiments. NS, not significant; Unpaired two-tailed Student’s *t*-test.

# **
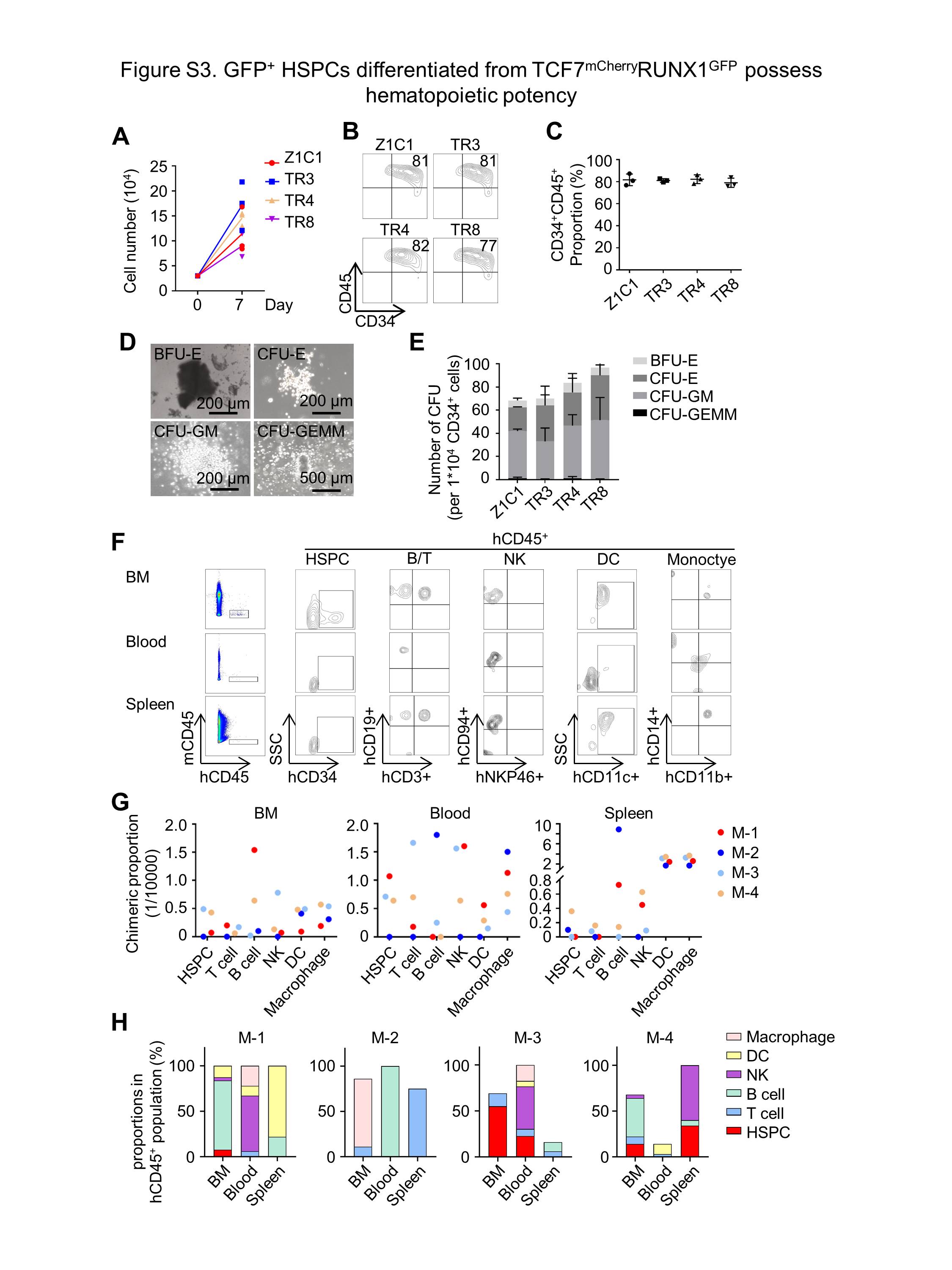
**

**Figure S3. GFP^+^ HSPCs differentiated from TR possess hematopoietic potency**

# **(A)** The GFP^+^ HSPCs differentiated from Z1C1, TR3, TR4, and TR8 underwent significant expansions in a 7 day *in vitro* culturing period. **(B)** The GFP^+^ HSPCs differentiated from Z1C1, TR3, TR4, and TR8 exhibited a dominant CD34^+^CD45^+^ phenotype after a 7 day *in vitro* expansion. **(C)** The proportions of CD34^+^CD45^+^ in the indicated expanded GFP^+^ HSPCs are presented. Data are shown as the mean ± SD from three independent experiments. **(D)** Representative phase contrast microscopy images of colony-formation unit assays. BFU-E, burst-forming unit-erythroid; CFU-E, colony forming unit-erythroid; CFU-G/M, colony forming unit-granulocytes, macrophages; CFU-GEMM, colony forming unit-granulocyte, erythrocyte, macrophages, megakaryocyte. **(E)** The statistical analysis of BFU-E, CFU-E, CFU-G/M, and CFU-GEMM formed by Z1C1, TR3, TR4, and TR8. Data are shown as the mean ± SD from three independent experiments. **(F)** The HSPCs derived from iPSCs maintain their population in the bone marrow of transplanted NOD/SCID mice and commit to both lymphoid and myeloid lineages. The human CD45^+^ populations are detected in the bone marrow, blood, and spleen of transplanted NOD/SCID mice. The B cells, NK cells, DC cells, and monocyte cells are detected in the bone marrow, blood, and spleen of transplanted NOD/SCID mice at varying levels. In addition, a population of cells expressing both CD19 and CD3 is detected in the bone marrow and spleen of transplanted mice. **(G)** Quantification of repopulated HSPCs as well as HSPC committed B, NK, DC, macrophages, and CD3^+^CD19^+^ cells in the bone marrow, blood, and spleen of HSPC transplanted NOD/SCID mice. M-1 to M-4 represent 4 NOD/SCID recipient mice transplanted with HSPCs. **(H)** Quantification of proportions of HSPCs as well as HSPC committed B, NK, DC, macrophages, and CD3^+^CD19^+^ cells in total grafted human CD45^+^ cells in the 4 NOD/SCID mice transplanted with HSPCs (M-1, M-2, M-3, and M-4).

# **
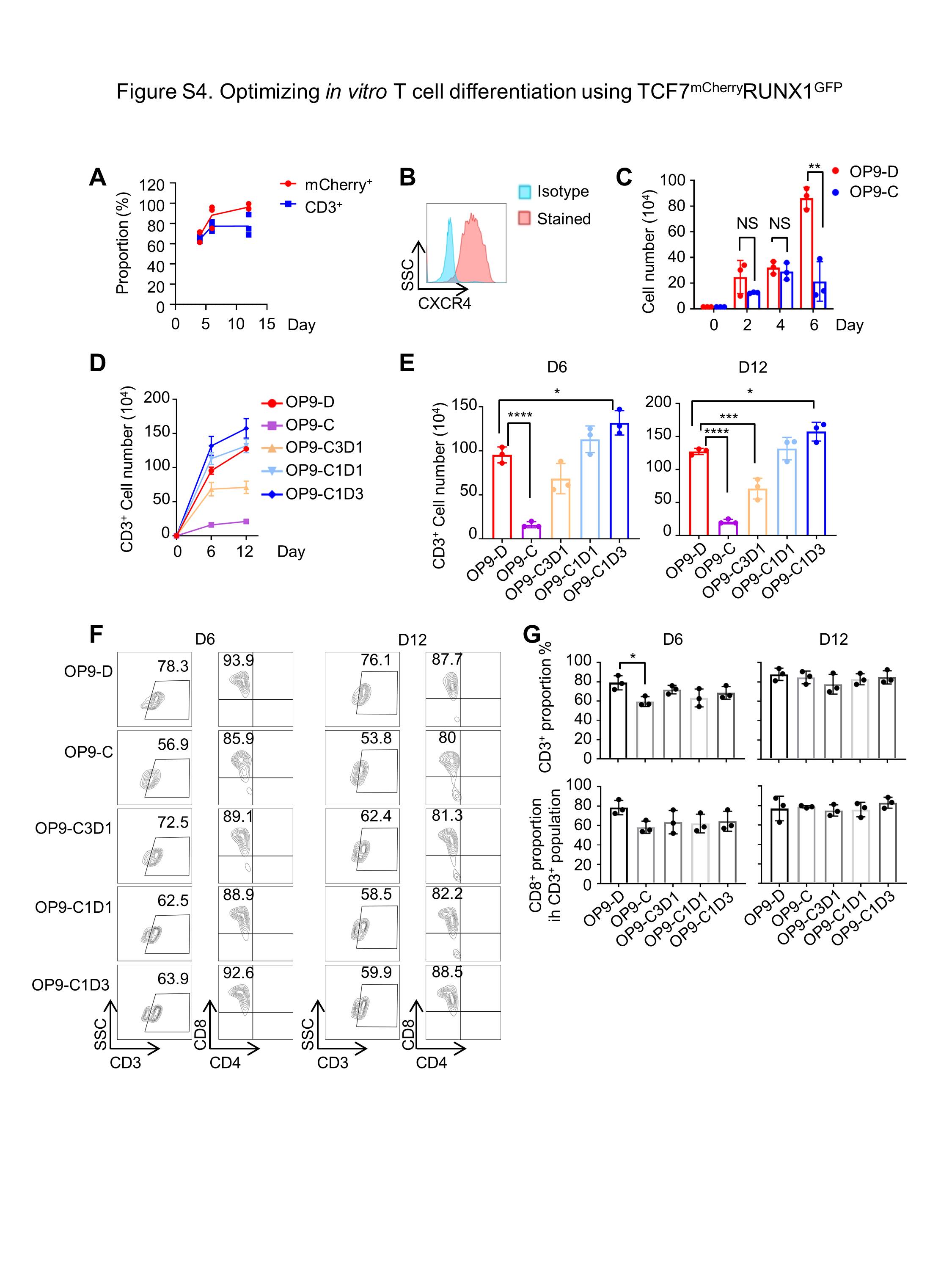
**

## Figure S4. Optimizing *in vitro* T cell differentiation using TCF7^mCherry^RUNX1^GFP^

**(A)** The proportions of mCherry^+^ and CD3^+^ populations in suspension cells at differentiating day 4, day 6, and day 12. **(B)** The T cells differentiated from TR4 iPSCs expressed CXCR4. **(C)** The quantification of suspension cells, differentiated from TR4 iPSC-committed HSPCs, was conducted on days 2, 4, and 6 using either the OP9-DL4 or OP9-CXCL12 feeder alone. Data are shown as the mean ± SD from three independent experiments. Unpaired two-tailed Student’s *t*-test. *P < 0.05, NS, not significant. **(D)** Quantification of CD3^+^ cells differentiated from TR4 iPSC committed HSPCs at differentiating day 6 or day 12 on the feeder of OP9-DL4 alone, OP9-CXCL12 alone, and mixtures of OP9-CXCL12 and OP9-DL4 at ratios of 3:1, 1:1, and 1:3. **(E)** The statistical analysis of the CD3^+^ cells during T cell differentiation in **D**. Data are shown as the mean ± SD from three independent experiments. Unpaired two-tailed Student’s *t*-test, *P < 0.05, ***P< 0.001, ****P< 0.0001, n=3. **(F)** Representative flow cytometry plots show the phenotype of T cells differentiated from TR4 iPSC committed HSPCs at differentiating day 6 or day 12 on the feeder of OP9-DL4 alone, OP9-CXCL12 alone, and mixtures of OP9-CXCL12 and OP9-DL4 at ratios of 3:1, 1:1, and 1:3. **(G)** The proportions of CD3^+^ cells in total differentiating cells as showed in **F** (up panel), and the proportions of CD8^+^ cells in each CD3^+^ populations as showed in **F** (down panel). Data are shown as the mean ± SD from three independent experiments. Unpaired two-tailed Student’s *t*-test, *P < 0.05. OP9-D, OP9-DL4; OP9-C, OP9-CXCL12; OP9-C3D1, OP9-CXCL12 mixed with OP9-DL4 at ratio of 3:1; OP9-C1D1, OP9-CXCL12 mixed with OP9-DL4 at ratio of 1:1; OP9-C1D3, OP9-CXCL12 mixed with OP9-DL4 at ratio of 1:3.

##
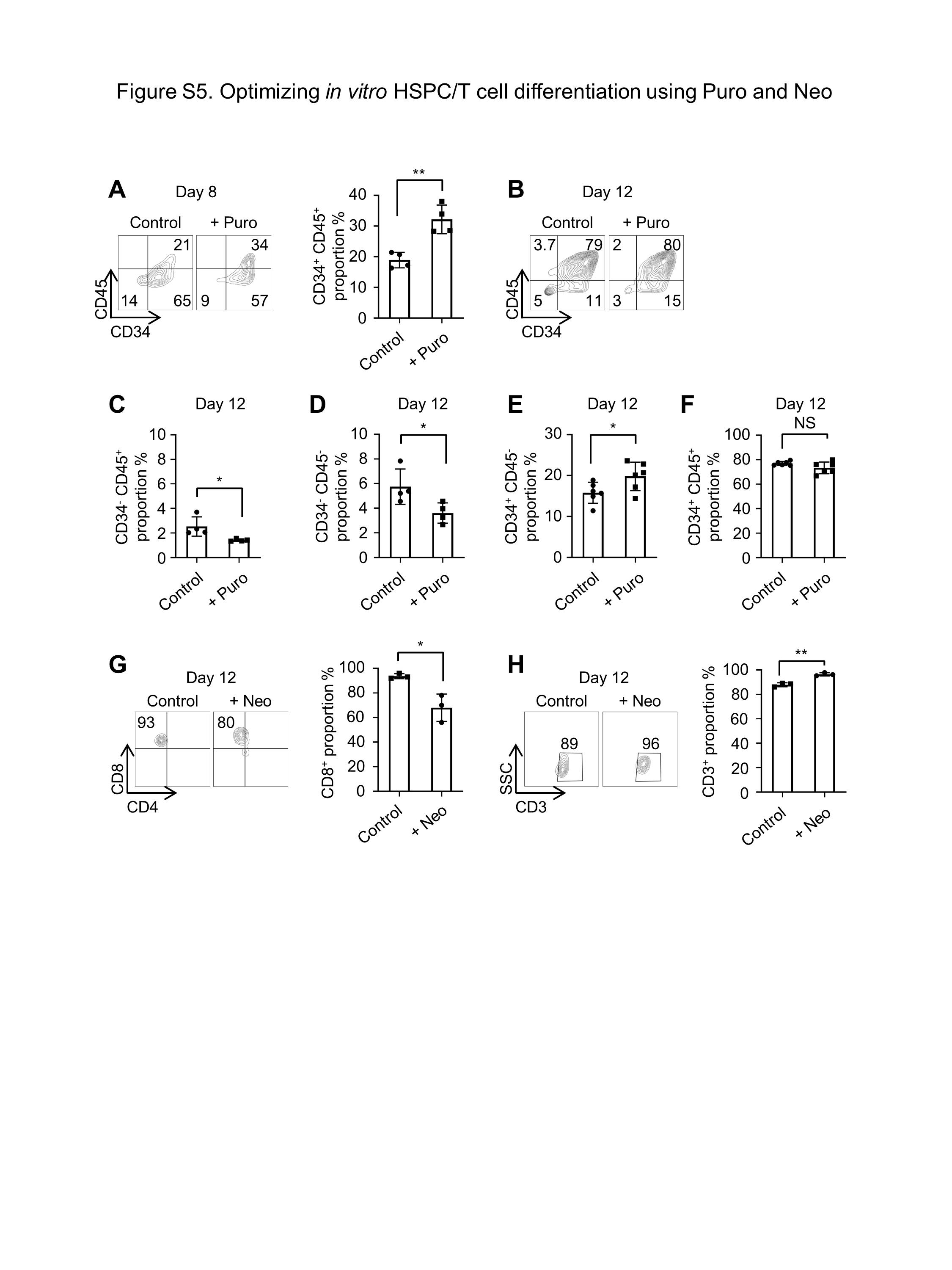


## Figure S5. Optimizing *in vitro* HSPC/T cell differentiation using Puro and Neo

**(A)** A higher proportion of CD34^+^CD45^+^ cells were collected on day 8, after a 3-day Puro treatment. Left, the representative flow cytometry plots illustrate the phenotype of HSPC cells that have differentiated from TR4 iPSCs. Right, statistical analysis of the proportions of CD34^+^CD45^+^ cells in suspension cells. Data are shown as the mean ± SD from four independent experiments. **P < 0.01; unpaired two-tailed Student’s *t*-test. **(B)** The representative flow cytometry plots show the phenotype of HSPC cells that have differentiated from TR4 iPSC on day 12. **(C-F)** Statistical analysis of the proportions of CD34^-^CD45^+^ cells **(C)**, CD34^-^CD45^-^ cells **(D)**, CD34^+^CD45^-^ cells **(E)** and CD34^+^CD45^+^ cells **(F)** in suspension cells. Data are shown as the mean ± SD from four to six independent experiments. *P < 0.05, NS, not significant; unpaired two-tailed Student’s *t*-test. **(G)** A lower proportion of CD8^+^ cells were collected on day 12, after a 6-day Neo treatment. Left, representative flow cytometry plots show the phenotype of CD8^+^ cells that have differentiated from TR4-HSPCs. Right, statistical analysis of the proportions of CD8^+^ cells in CD3^+^ cells. Data are shown as the mean ± SD from three independent experiments. *P < 0.05; unpaired two-tailed Student’s *t*-test. **(H)** A higher proportion of CD3^+^ cells were collected on day 12, after a 6-day Neo treatment. Left, representative flow cytometry plots show the phenotype of T cells that have differentiated from TR4-HSPCs. Right, statistical analysis of the proportions of CD3^+^ cells in suspension cells. Data are shown as the mean ± SD from three independent experiments. **P < 0.01; unpaired two-tailed Student’s *t*-test.

##
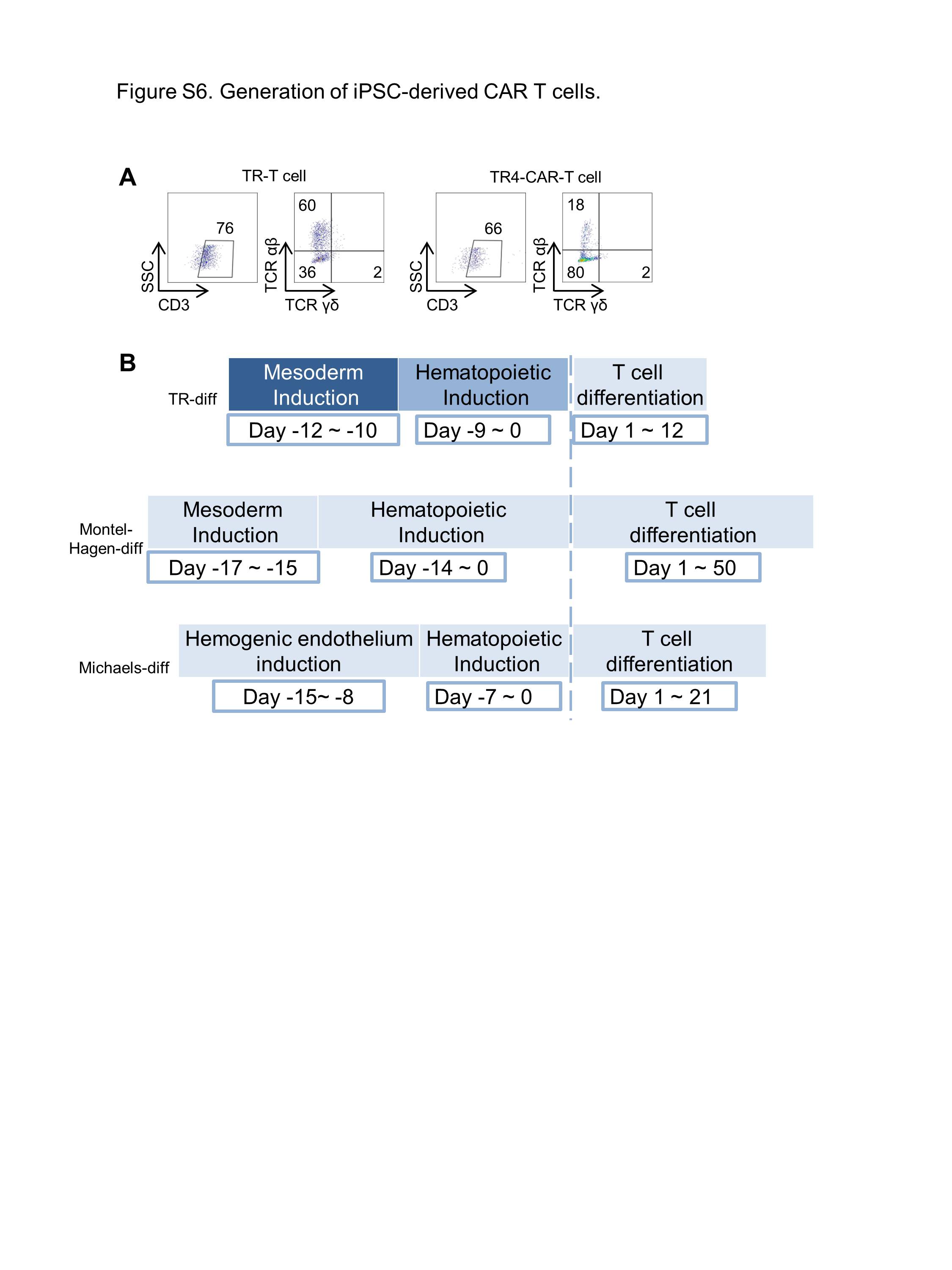


## Figure S6. Characteristics of CAR-T cells differentiated from TCF7^mCherry^RUNX1^GFP^

**(A)** The representative flow cytometry analysis illustrates the expression of TCRαβ and TCRγδ on TR-T cells and TR4-CAR-T cells. **(B)** Comparison of different induction schemes for T cell production from iPSCs. TR-diff: differentiation system in our study. Montel-Hagen-diff: differentiation system published in *Cell Stem Cell* at 2019. Michaels-diff: differentiation system published in *Science Advances* at 2022.
